# Supplementary material for: Potential mechanisms and drug prediction of Rheumatoid Arthritis and primary Sjögren’s Syndrome: A public databases-based study
Source: PLoS One. 2024 Feb 15;19(2):e0298447. doi: 10.1371/journal.pone.0298447 (PMC10868835; doi:10.1371/journal.pone.0298447)
Supplement: S1 File — (DOCX) [file pone.0298447.s001.docx]

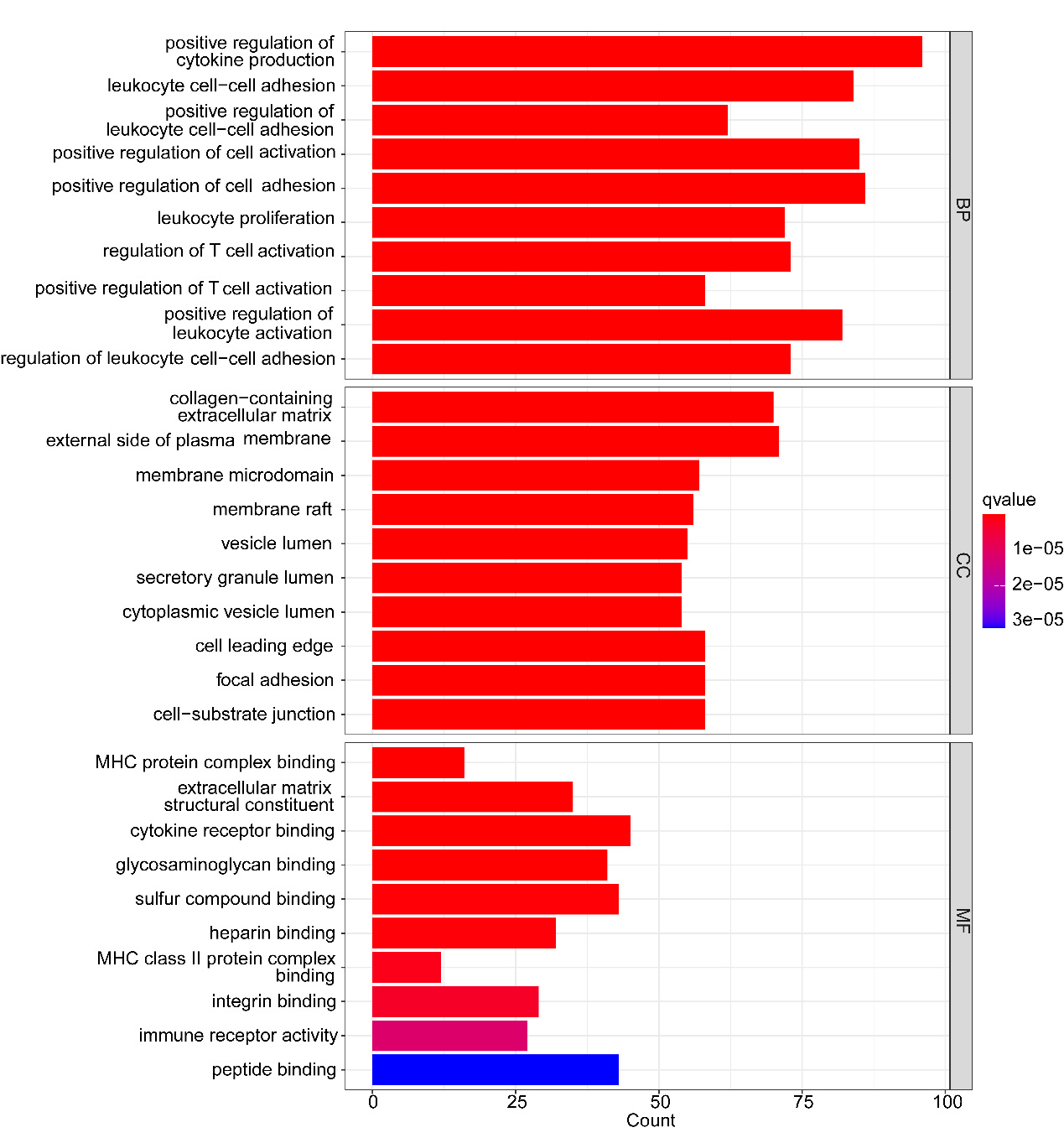


**S1 Figure. GO enrichment analysis of the positively related modules in RA.**


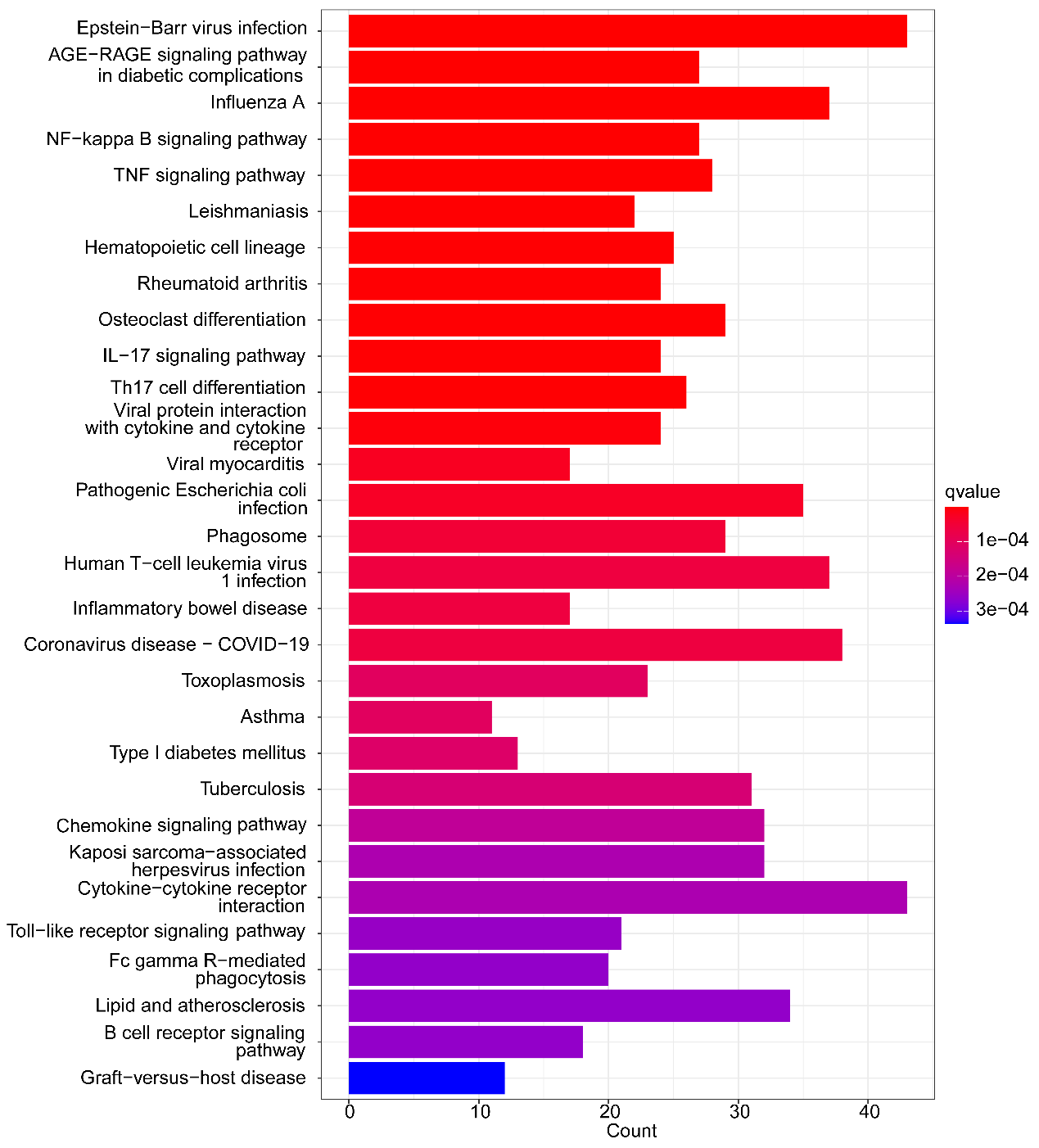


**S2 Figure. KEGG enrichment analysis of the positively related modules in RA.**


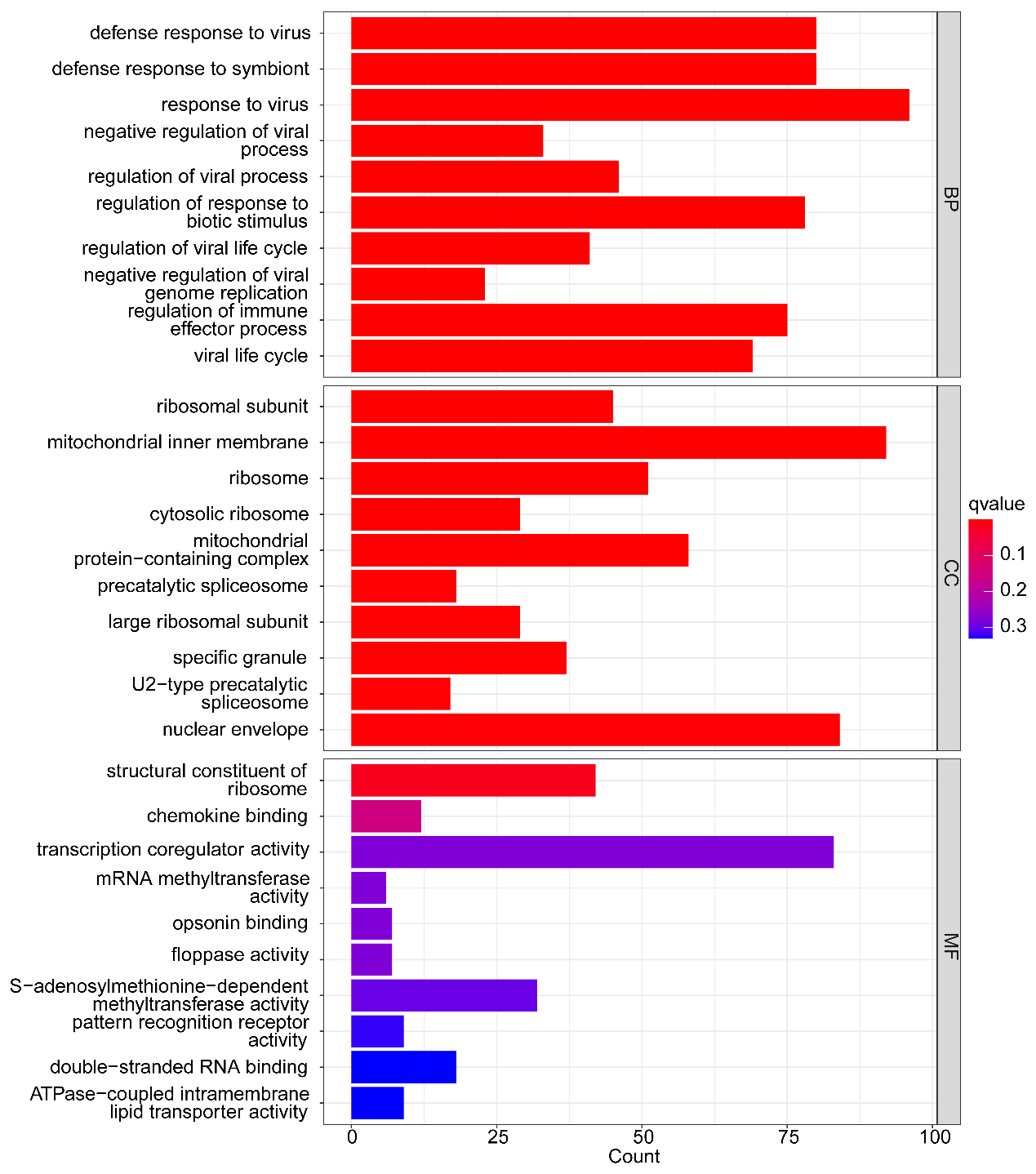


**S3 Figure. GO enrichment analysis of the positively related modules in pSS.**


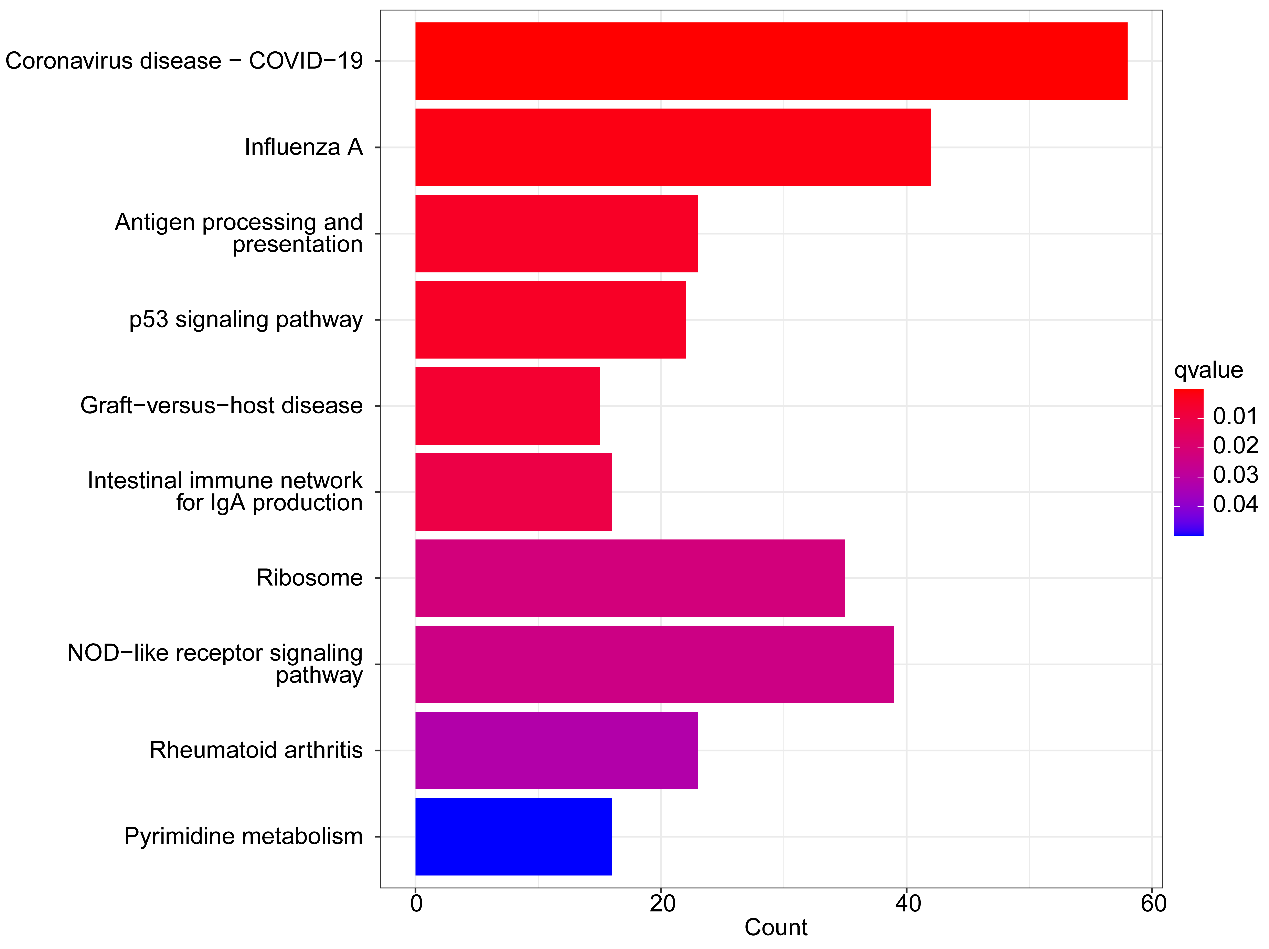


**S4 Figure. KEGG enrichment analysis of the positively related modules in pSS.**

**S1 Table. Common targets of RA and pSS in GeneCards and CTD databases.**

| 322 Common targets of RA and pSS in GeneCards and CTD databases | | | | | | | | |
| --- | --- | --- | --- | --- | --- | --- | --- | --- |
| IL6 | IL10 | TNF | STAT4 | MIF | IRF5 | IL2RA | IL2RB | TLR1 |
| CXCL8 | IL2 | IL18 | TLR4 | CCL2 | FAS | IL4 | IL23R | IL1A |
| FASLG | TTR | SPP1 | FCGR3B | SERPINH1 | TNIP1 | ICAM1 | CSF2 | IL13 |
| ALB | HLA-DPB1 | IL23A | FSTL1 | PRTN3 | PTGS1 | HSPD1 | MPO | CCL3 |
| S100A8 | CXCL12 | MICB | HSPA5 | TFRC | TLR9 | JUN | TRAF6 | ITGB2 |
| CRH | CD4 | CCR7 | CCL4 | ALOX5 | TNFRSF10A | NFKB1 | CD86 | SELL |
| HLA-A | IL12A | CALCA | PSMB9 | GSTM1 | CCL21 | INS | HMGB2 | TLR3 |
| ACE | GPT | TNFSF4 | TREX1 | DNASE1 | MYC | ICOSLG | RELA | PON1 |
| HLA-G | CCN1 | CD8A | HIF1A | IGF1 | POMC | XDH | IL1RL1 | FN1 |
| CTLA4 | CRP | CD247 | TNFRSF1A | IL17A | MMP3 | IFNG | IL1RN | BCL2L1 |
| CD40 | MMP9 | TLR2 | VEGFA | TGFB1 | VCAM1 | CCL5 | S100A9 | CXCR2 |
| BGLAP | TNFAIP3 | CD40LG | SELE | CD79A | HMGB1 | CXCL10 | ADAM17 | ATM |
| IFNB1 | NFKBIA | CD28 | MAPK1 | NOS2 | CD44 | HSPA4 | CD36 | C3 |
| MAPK8 | CXCL1 | CXCR4 | CCL20 | CR1 | NR4A2 | NR3C1 | ELANE | APOE |
| CTSG | ITGAL | LTBR | IL12B | PRKCD | IRAK1 | VDR | IL22RA1 | CASP3 |
| TPMT | MYD88 | TP53 | LTF | PRL | VWF | ADA | IL3 | FOS |
| TRAF3IP2 | PPARG | CD58 | BCL2 | IRF4 | ESR1 | NOS3 | MS4A1 | TLR7 |
| VIM | IGHM | CD274 | TNFSF13 | P2RX7 | IRF1 | CALR | HSPA8 | ITGA4 |
| TNFSF10 | ITGB1 | SOCS3 | PTEN | F3 | IL7R | NPY | GSTT1 | KITLG |
| SOD2 | PRDM1 | F2RL1 | EDN1 | GZMA | TAC1 | CD55 | PLAT | KLRK1 |
| NGF | PSMB8 | EGFR | CST3 | CD2 | PARP1 | F2 | IL21R | PDPN |
| KIR2DS2 | COL1A2 | CYP19A1 | SPTAN1 | NFATC1 | CCR8 | CD1D | THBS1 | GTF2I |
| XBP1 | TYRO3 | ALPP | IRF3 | CLU | AIM2 | HSPG2 | MAP2K4 | BAX |
| CXCR6 | BCL6 | ENO2 | CYP1A1 | FPR2 | CYP17A1 | CYP2D6 | CYP3A4 | LCN2 |
| VTCN1 | PF4 | CYCS | CDKN3 | SPHK1 | CCL3L1 | STAT5A | BST2 | SDC1 |
| BAK1 | CASP9 | EZR | CCK | AGTR1 | IRF7 | ITGA1 | RPLP1 | MALT1 |
| ABCA1 | ADIPOR1 | LILRA3 | GAPDH | AQP5 | CTSD | PLA2G7 | AREG | CCR9 |
| PSME3 | HSPB1 | TPI1 | NCF1 | RECK | XRCC5 | PRLR | EIF2AK3 | GFAP |
| CTSS | ADIPOR2 | MX1 | TOP1 | ID3 | DDIT3 | NOS1 | CEACAM5 | MUC16 |
| MKI67 | TRIM63 | ITPR3 | FST | ARHGAP45 | H1-5 | OAS1 | LEF1 | INSR |
| EPX | FUT7 | NR3C2 | COL4A3 | ITGA3 | MOG | CYP4F3 | TCP1 | ITPR1 |
| HNRNPH1 | PLA1A | TFAP2A | DLAT | BCL10 | RORA | NCL | APAF1 | LAMB1 |
| LPO | TNRC6A | IL1B | TNFRSF11B | STAT3 | ADIPOQ | RETN | CTSB | STAT1 |
| CCN2 | CFLAR | CCL11 | HRH2 | CXCL11 | CD27 | SELP | CASP8 | ITGA2 |
| TRIM21 | AKT1 | THBD | TNFRSF17 | CA2 | MBP | CHRM3 | TGFA | PCNA |
| MYB | F2R | ANXA11 | GADD45A | GAD1 | CD93 | ADCYAP1 |  |  |

**S2 Table. The potential drugs of hub genes using DGIbd database.**

| \| search_term \| match_term \| match_type \| gene \| drug \| \| --- \| --- \| --- \| --- \| --- \| \| PSMB9 \| PSMB9 \| Definite \| PSMB9 \| CARFILZOMIB \| \| PSMB9 \| PSMB9 \| Definite \| PSMB9 \| OPROZOMIB \| \| PSMB9 \| PSMB9 \| Definite \| PSMB9 \| BORTEZOMIB \| \| PSMB9 \| PSMB9 \| Definite \| PSMB9 \| MARIZOMIB \| \| PSMB9 \| PSMB9 \| Definite \| PSMB9 \| IXAZOMIB CITRATE \| \| CXCL10 \| CXCL10 \| Definite \| CXCL10 \| ELDELUMAB \| \| CXCL10 \| CXCL10 \| Definite \| CXCL10 \| RITONAVIR \| \| CXCL10 \| CXCL10 \| Definite \| CXCL10 \| NI-0801 \| \| CXCL10 \| CXCL10 \| Definite \| CXCL10 \| ATROPINE \| \| CXCL10 \| CXCL10 \| Definite \| CXCL10 \| ANTIBIOTIC \| \| CXCL10 \| CXCL10 \| Definite \| CXCL10 \| ATORVASTATIN \| \| CXCL10 \| CXCL10 \| Definite \| CXCL10 \| REGRAMOSTIM \| \| CXCL10 \| CXCL10 \| Definite \| CXCL10 \| METHYLPREDNISOLONE \| \| CXCL10 \| CXCL10 \| Definite \| CXCL10 \| OXALIPLATIN \| \| CXCL10 \| CXCL10 \| Definite \| CXCL10 \| TESTOSTERONE \| \| CXCL10 \| CXCL10 \| Definite \| CXCL10 \| STAVUDINE \| \| CXCL10 \| CXCL10 \| Definite \| CXCL10 \| ZIDOVUDINE \| \| ITGA4 \| ITGA4 \| Definite \| ITGA4 \| CHEMBL88478 \| \| ITGA4 \| ITGA4 \| Definite \| ITGA4 \| VEDOLIZUMAB \| \| ITGA4 \| ITGA4 \| Definite \| ITGA4 \| NATALIZUMAB \| \| ITGA4 \| ITGA4 \| Definite \| ITGA4 \| ABRILUMAB \| \| ITGA4 \| ITGA4 \| Definite \| ITGA4 \| FIRATEGRAST \| \| ITGA4 \| ITGA4 \| Definite \| ITGA4 \| SENKTIDE \| \| GZMA \| GZMA \| Definite \| GZMA \| CYCLOSPORINE \| |
| --- | --- | --- | --- | --- | --- | --- | --- | --- | --- | --- | --- | --- | --- | --- | --- | --- | --- | --- | --- | --- | --- | --- | --- | --- | --- | --- | --- | --- | --- | --- | --- | --- | --- | --- | --- | --- | --- | --- | --- | --- | --- | --- | --- | --- | --- | --- | --- | --- | --- | --- | --- | --- | --- | --- | --- | --- | --- | --- | --- | --- | --- | --- | --- | --- | --- | --- | --- | --- | --- | --- | --- | --- | --- | --- | --- | --- | --- | --- | --- | --- | --- | --- | --- | --- | --- | --- | --- | --- | --- | --- | --- | --- | --- | --- | --- | --- | --- | --- | --- | --- | --- | --- | --- | --- | --- | --- | --- | --- | --- | --- | --- | --- | --- | --- | --- | --- | --- | --- | --- | --- | --- | --- | --- | --- | --- |
