# Supplementary material for: Potential mechanisms and drug prediction of Rheumatoid Arthritis and primary Sjögren’s Syndrome: A public databases-based study
Source: PLoS One. 2024 Feb 15;19(2):e0298447. doi: 10.1371/journal.pone.0298447 (PMC10868835; doi:10.1371/journal.pone.0298447)
Supplement: Attachment — Submitted filename: Response to Reviewers.docx [file pone.0298447.s002.docx]

Dear editors and reviewers:

Thank you very much for your careful review of our manuscript entitled “Potential mechanisms and drug prediction of Rheumatoid Arthritis and primary Sjögren’s Syndrome: a public databases-based study”. We have carefully considered the reviewers’ professional comments and made a point to point response as follows:

**To Reviewer 1：**

1. Regarding the manuscript entitled “Potential mechanisms and drug prediction of Rheumatoid Arthritis and primary Sjögren’s Syndrome : a public databases-based study” . The manuscript is interesting, but the quality of the figures should be improved.

**Response：**Thank you for your professional suggestions. Later, I will re-upload high-resolution figures in the manuscript that meet the requirements of the magazine.

**To Reviewer 2：**

1. Please provide the flowchart of study in a separate figure.

**Response：**Thank you for your professional suggestions. Fig 1 in the manuscript is the flow chart of this study, and I will upload a separate flow chart again in the system.

2. In the introduction section please provide brief description about WGCNA method. Reading the paper PMID: 37636389 is suggested.

**Response:** Thank you for your professional suggestions. We describe the WGCNA approach in the introduction section. Weighted Gene Co-expression Network Analysis (WGCNA) is a computational biology method used to explore patterns of gene correlations among different samples. It can be used to cluster gene modules that exhibit highly coordinated changes in various biological processes and correlate these gene modules with clinical feature data [1, 2]. By analyzing the results, important core modules and molecules can be identified. The introduction of co-expression networks has facilitated a network-based gene selection method, which can be used in basic medical research to identify specific candidate biomarkers [3] or immunotherapy targets [4].

The main computational process of WGCNA involves raising correlation values between genes to a power, resulting in a topological overlap matrix network that better fits the characteristics of a scale-free network [5]. Its characteristic is the strengthening of strong correlations and weakening of weak correlations. In this study, the expression profile data of RA and pSS were analyzed using the WGCNA analysis method. After calculating and analyzing the correlation between gene modules and clinical feature data, the relationship between each gene module and clinical features was clarified. Combined with the analysis of differentially expressed genes, the biological functions and target genes of RA and pSS were identified.

**References**

1.	Zhang T, Wong G. Gene expression data analysis using Hellinger correlation in weighted gene co-expression networks (WGCNA). Computational and structural biotechnology journal. 2022;20:3851-63.

2.	Liu K, Chen S, Lu R. Identification of important genes related to ferroptosis and hypoxia in acute myocardial infarction based on WGCNA. Bioengineered. 2021;12(1):7950-63.

3.	Ye H, Wang R, Wei J, Wang Y, Zhang X, Wang L. Bioinformatics Analysis Identifies Potential Ferroptosis Key Gene in Type 2 Diabetic Islet Dysfunction. Frontiers in endocrinology. 2022;13:904312.

4.	Afshar S, Leili T, Amini P, Dinu I. Introducing novel key genes and transcription factors associated with rectal cancer response to chemoradiation through co-expression network analysis. Heliyon. 2023;9(8):e18869.

5.	Langfelder P, Horvath S. WGCNA: an R package for weighted correlation network analysis. BMC Bioinformatics. 2008;9:559.

3. "GSE55235, GSE55457, GSE1919, GSE110169, GSE84844, GSE23117, GSE40611, and GSE84844" are data series not data set please revise related sentences.

**Response：**Thank you for your professional suggestions. Finally, we downloaded the data series numbered GSE55235, GSE55457, GSE1919, GSE110169, GSE84844, GSE23117, GSE40611, and GSE84844.

4. In the senetence "The co-expression network was built using microarray dataset GSE55235 and dataset GSE84844 to obtain the RA and pSS-associated modules" did the authors perform WGCNA on selected genes of data series . How did they screen genes for WGCNA?

**Response**：Thank you for your professional suggestions. We performed WGCNA on selected genes of data series GSE55235 and GSE84844. We used the R package “WGCNA” to identify disease trait-related modules and hub genes. First, the gene expression data was clustered to determine the optimal soft threshold (β) based on the average connectivity and scale-free topology fit index (R2). Multiple gene modules and hierarchical clustering trees were constructed. Then, Pearson correlation analysis was performed between genes to obtain a relationship matrix, and the module with the highest correlation was selected as the key gene module.

Based on the criteria of a soft-thresholding power of 10 (scale free R2 = 0.85), a cut height of 0.25, and a minimum module size of 50, calculations were made to achieve scale-free topology for the dataset GSE55235. For the dataset GSE84844, we specified a soft-thresholding power of 4, a cut height of 0.4, and a minimum module size of 50 to confirm the co-expression modules. The genes selected for further analysis were those with strong correlation coefficients with clinical characteristics. We identified key modules in RA and pSS using Pearson correlation coefficients and the p-value of each eigengene and disease trait. Afterward, the genes in these key modules that were positively associated with RA and pSS were identified as shared genes.

5. In sentence "An adjusted p < 0.05 in GO terms or KEGG pathways was considered statistically significant and visualized using the “GOplot” package " please mention GOplot package under R software.

**Response：**Thank you for your professional suggestions. An adjusted p < 0.05 in GO terms or KEGG pathways was considered statistically significant and visualized using the “GOplot” package under R 4.2.1 version.
